# Gastrointestinal manifestations in children with COVID-19 infection: Retrospective tertiary center experience

**DOI:** 10.3389/fped.2022.925520

**Published:** 2022-12-21

**Authors:** Rana R Bitar, Bushra Alattas, Amer Azaz, David Rawat, Mohamad Miqdady

**Affiliations:** Sheikh Khalifa Medical City, Abu Dhabi, United Arab Emirates

**Keywords:** COVID-19 in children, COVID-19 clinical manifestation, COVID-19 infection, COVID-19 liver injury, COVID-19 complication

## Abstract

**Objective:**

The majority of pediatric severe acute respiratory syndrome coronavirus 2 (COVID-19) cases demonstrate asymptomatic, mild or moderate disease. The main symptoms in children with COVID-19 are respiratory symptoms but some patients develop gastrointestinal symptoms and liver injury. We aim to review gastrointestinal symptoms and liver injury in children with confirmed COVID-19 infection.

**Method:**

This is a retrospective case note review of children with positive COVID-19 nasal Polymerase Chain Reaction aged 0–18 years admitted to a tertiary pediatric hospital from March 1st till June 1st 2020.

**Results:**

180 children were identified. Mean age was 5 years (Range: 0.01–17), the majority of patients were school aged (30%). Patients were mainly from East Asia 81 (45%) and Arabs 67 (37%). Gastrointestinal symptoms were encountered in 48 (27%) patients and 8 (4%) patients had only Gastrointestinal symptoms with no associated fever or respiratory symptoms. Liver injury was seen in 57 (32%) patients. Patients with fever and cough were more likely to have gastrointestinal symptoms (*P* = <0.001 and 0.004 respectively). Fever was more likely to be associated with liver injury (*P* = 0.021). Children with abdominal pain were more likely to have elevated C-Reactive Protein (*P* = 0.037). Patients with diarrhea and vomiting were more likely to have elevated procalcitonin (*P* = 0.034 and 0.002 respectively). Children with Gastrointestinal symptoms were not more likely to be admitted to Pediatric Intensive Care Unit (*P* = 0.57).

**Conclusion:**

COVID-19 infection in children can display gastrointestinal symptoms at initial presentation. Additionally, gastrointestinal symptoms can be the only symptoms patients display. We demonstrated that children with gastrointestinal symptoms and liver injury can develop more severe COVID-19 disease and are more likely to have fever, cough, and raised inflammatory markers. Identifying children with gastrointestinal manifestations needs to be part of the initial screening assessment of children.

What is known?

• Pediatric COVID-19 cases mostly demonstrate asymptomatic, mild or moderate disease.

• The symptoms in children are mainly respiratory but some display gastrointestinal symptoms.

• Children with COVID-19 display increased gastrointestinal symptoms when compared to adults.

What is new?

• Children with COVID-19 displaying gastrointestinal symptoms are more likely to have fever, cough and elevated inflammatory markers.

• Children with liver injury are more likely to develop fever.

• Children with gastrointestinal involvement in COVID-19 are more likely to demonstrate more severe disease but are not more likely to be admitted to PICU.

## Introduction

Severe acute respiratory syndrome coronavirus 2 was the cause of a series of cases with severe pneumonia initially reported in Wuhan, China (1, 2) declared as COVID-19 by the WHO. 2.1%–5% of infected cases are children (3, 4). The pediatric population display a mild disease and majority (over 90%) have mild, moderate or asymptomatic disease (5–8) Approximately 1% of children develop severe disease requiring admission to intensive care unit (9).

The typical presentation of COVID-19 includes fever, weakness, nausea, and pulmonary symptoms such as dry cough and dyspnea. A proportion of affected patients also have digestive manifestations, such as anorexia, nausea, vomiting, diarrhea, and abdominal pain. In addition, liver injury is well described in children with COVID-19 infection. Viral fecal shedding for several weeks after diagnosis has been reported (10), COVID-19 virus was observed in rectal swabs in eight out of ten children after nasopharyngeal swabs returned negative (11). The viral shedding in stool and nasal secretions make children possible facilitators of viral transmission (5) and is one of the possible explanations for the prevalence of Gastrointestinal (GI) symptoms in CVOID-19 infected children.

GI symptoms are reported to range from 12% to 21% in the pediatric literature with varying frequencies in the United States, Europe and China (11–13). In addition, gastrointestinal symptoms are observed more frequently in children with younger age and fever (14). Children with GI symptoms show higher levels of C-reactive protein and procalcitonin this suggests that more severe COVID disease is observed with GI symptoms in children (15).

The United Arab Emirates (UAE) developed a strong testing program. They performed Polymerase Chain Reaction (PCR) nasal swab tests of 2,850 per 100,000 population per day (11), achieving identification and isolation of most paediatric COVID-19 infected children. We aim to review GI symptoms and liver injury in children with COVID-19, to evaluate risk factors predisposing for GI symptoms, and to assess if GI symptoms and liver injury are associated with more severe COVID-19 disease in our population.

## Method

Approval from the Institutional Review Board Committee for COVID-19 research in the Department of Health, Abu Dhabi was obtained. We retrospectively reviewed the electronic medical records of patients with COVID-19 diagnosed based on nasal swab PCR aged 0–18 years old from 1st of March to 1st of June 2020. This study was conducted at the start of the pandemic. At the start of the pandemic all paediatric patients were admitted to hospital, Sheikh Khalifa Medical City (the only designated pediatric COVID-19 Hospital in Abu Dhabi City for children) to ensure appropriate isolation measures are carried out and the medical needs of the children are met. Patients were reviewed by a pediatrician daily and were discharged if they had two negative COVID-19 PCR test 48 h apart and were medically fit for discharge.

This patient sample has been utilized in another publication, British Medical Journal, Paediatrics open 2021, Clinical manifestation and outcome in children with COVID-19 infection in Abu Dhabi: a retrospective single centre study.

We collected patient demographics, ethnicity, Body Mass Index (BMI), length of admission, background clinical conditions, symptoms at presentation, biochemical markers, complications and mortality. Presenting symptoms reviewed were fever, cough, abdominal pain, diarrhea, and vomiting. Biochemical markers included C-reactive protein (CRP), procalcitonin, lymphocyte count, Alanine aminotransaminase (ALT) and Aspartate aminotransferase (AST). Patients with temperature >37.8 °C were considered to have fever, liver injury was defined as an increase in either AST or ALT or both above normal level for age and sex. Elevated CRP and Procalcitonin were defined as an increase above normal level for age. Lymphopenia was defined as a lymphocyte count below normal level for age and sex. Patients were classified as underweight if BMI was <18.5 kg/m^2^, healthy if BMI was between 18.5 and 25 kg/m^2^, overweight if BMI was 25 to 30 kg/m^2^ and obese if BMI is >30 kg/m^2^. All patients were followed up for 1 year for COVID related complications.

## Statistical analysis

The Statistical Package for Social Sciences version 21.0 for Windows (SPSS Inc., Chicago, IL, USA) was used. Categorical variables are presented as frequency and percentage, while numerical variables are presented as mean ± standard deviation (SD), and or median (centile) with range. Correlation between categorical variables was analyzed using chi-square or Fisher's exact test, as appropriate. Comparison of non-normally distributed numerical variables was carried out using nonparametric tests including median test. A *p* value of <0.05 was considered to reject the null hypothesis. Sex, age, ethnicity, BMI, background medical disease, cough and fever were analyzed using multiple regression analysis to evaluate risk factors predisposing to gastrointestinal symptoms. For modeling binary outcomes, (GI complications, No GI complications), we transformed the fitted value from a linear regression to FIT in between 0 and 1. One can interpret it as a probability of a match conditional on the regressor value.

## Results

**Figure 1:**
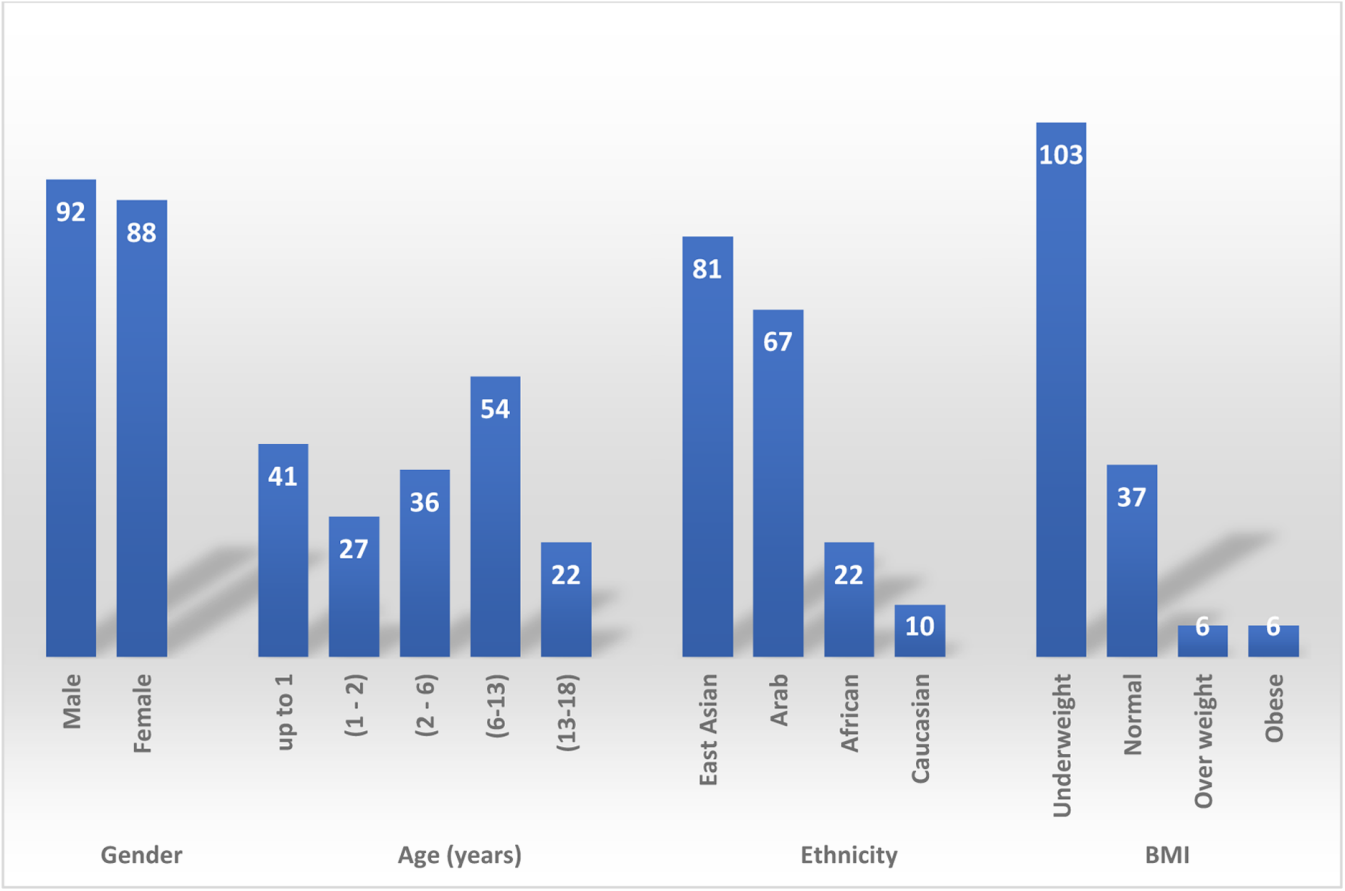
Patient demographics.

180 children were identified. Patient demographic is described in Figure 1. 92 (51%) patients were males, mean age was 5 years (Range: 0.01–17). The majority of patients, 54 (30%) were school aged, 6–13 years old. Followed by infants, <1 year, 41 (23%) patients. Patients were mainly from East Asia, 81 (45%) patients, and Arab patient were the second common ethnicity, 67 (37%) patient. BMI was only checked for 152 patients, most patients 103 (67%) were underweight, 37 (25%) patients had normal BMI, there were 12 (8%) patients with BMI > 25 kg/m^2^ and of these 12 patients, six were obese.

**Figure 2:**
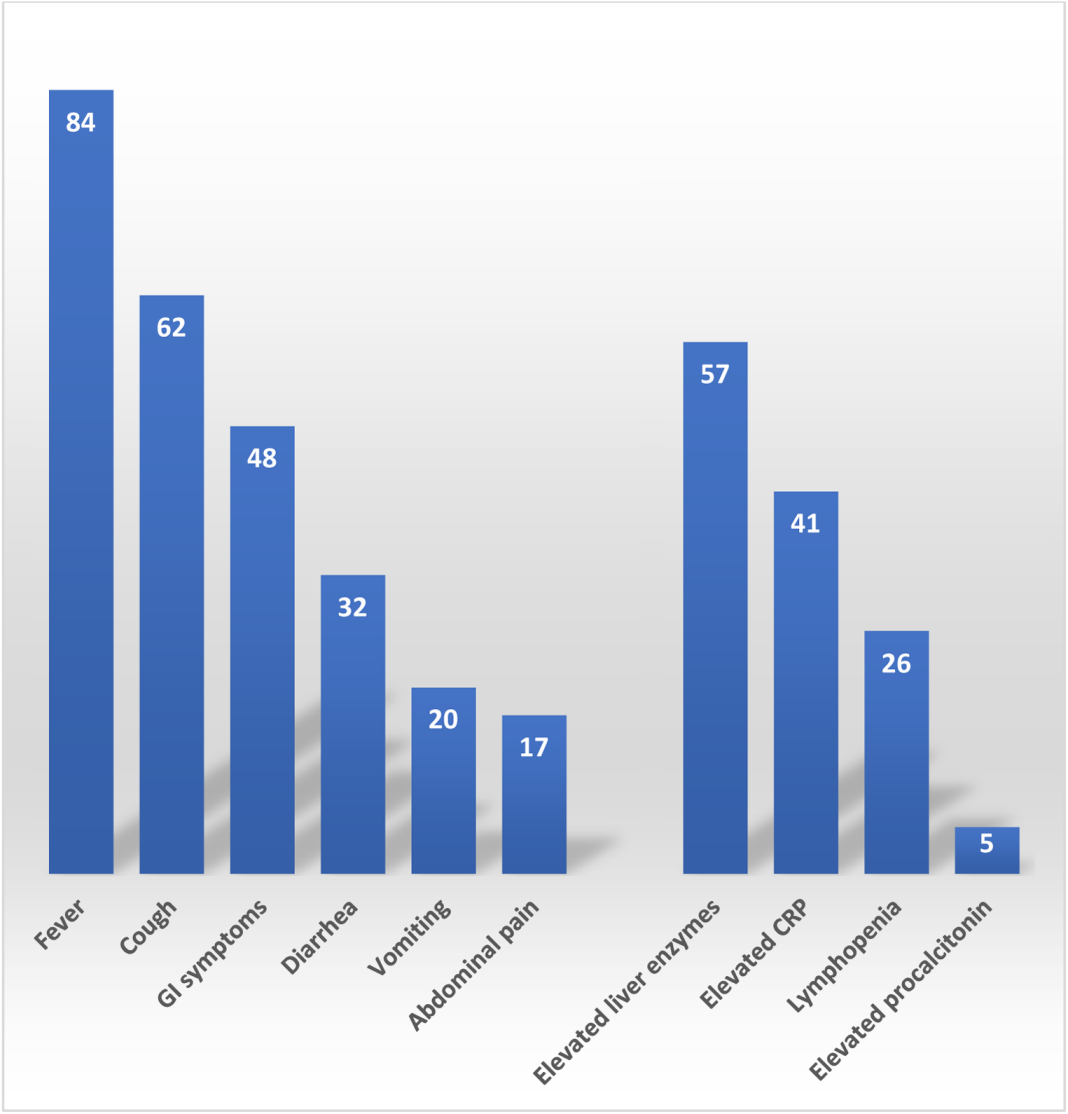
Clinical data.

Clinical presentation included; fever in 84 (47%) patients, 62 (34%) patients with cough, 48 (27%) encountered GI symptoms, 32 (18%) presented with diarrhea, 20 (11%) patients suffered from vomiting, and 17 (9%) had abdominal pain (Figure 2). Interestingly, 8 (4%) patients only had GI symptoms with no associated fever of respiratory symptoms. The most common inflammatory marker to be elevated was CRP, 41(23%) had elevated CRP, lymphopenia was only seen in 26 (14%) patients and only 5 (3%) patients had elevated procalcitonin. Liver injury in the form of elevated ALT and/or AST was seen in 57 (34%) patients. Median ALT was 22 IU/L (Rang: 5–220), Median AST was 46 IU/L (Rang: 31–359).

Sex, age, ethnicity, BMI, background medical disease, cough and fever were analyzed using multiple regression analysis to evaluate risk factors predisposing to gastrointestinal symptoms (Table 1). Children with fever and cough were more likely to have GI symptoms (*P* = <0.001 and 0.004 respectively). Sex, age, ethnicity, BMI, and patient having a background medical disease did not predispose to the development of GI symptoms. Children with abdominal pain were more likely to have elevated CRP (*P* = 0.037). Patients with diarrhea and vomiting were more likely to have elevated procalcitonin (*P* = 0.034 and 0.002 respectively). Patients with fever were also more likely to have liver injury (*P* = 0.021).

**Table 1:** Regression analysis, predisposing factors to development of GI symptoms.

	Unstandardized Coefficients		Standardized Coefficients	t	*P* Value
	B	Std. Error	Beta		
(Constant)	1.678	.155		10.824	.000
Sex	−.059	.117	−.067	−.503	.616
Ethnicity	−.133	.063	−.330	−2.121	.035
Age	.070	.081	.209	.856	.393
BMI	−.006	.037	−.020	−.160	.873
Medical disease	−.053	.158	−.055	−.339	.735
Cough	−.452	.155	−.486	−2.916	.004
Fever	.745	.101	.840	7.407	.000
Dependant Variable: GI symptoms					
R^2 ^= .282 F = 9.668 N = 180					

**Figure 3:**
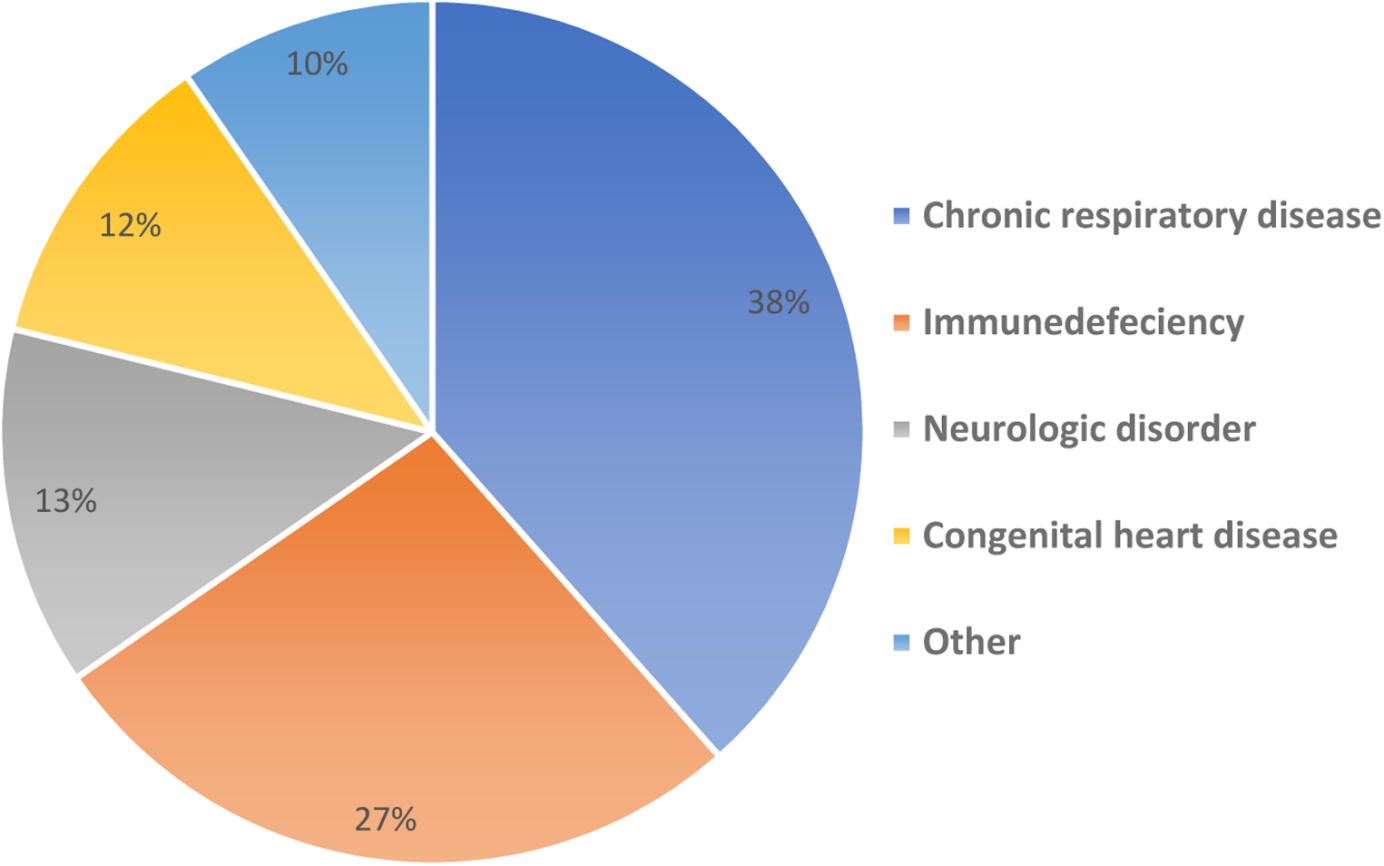
Background medical disease.

Overall, 52 (29%) had comorbid medical diseases (Figure 3) including one of the patients with multiple organ transplantation; liver, bowel and pancreas transplant, this patient developed post COVID small intestinal rejection 10 days post COVID infection. The median length of hospital stay was 6 days (Range: 0–60). The patients were allowed to be discharged to the community if they had two COVID negative PCR tests 48 h apart. Six (3%) patients were admitted to Pediatric Intensive Care Unit (PICU) and there was no reported mortality. Of the six patients admitted to PICU; only one patient had GI symptoms, and only one patient was obese with BMI of 33.68 kg/m^2^. Children with GI symptoms were not more likely to be admitted to PICU (*P* = 0.57).

## Discussion

Respiratory illness is the main presentation of Children with COVID-19 disease and GI symptoms can also be present. GI symptoms had been reported in 24.8% of paediatric patients (16). 27% of patients in our review experienced GI symptoms. GI manifestations in COVID-19 includes; diarrhea (2%–50%), anorexia (40%–50%), vomiting (4%–67%), nausea (1%–30%) and abdominal pain (2%–6%). Diarrhea and vomiting are the most common GI symptoms described (17). We observed diarrhea in 18%, vomiting in 11% and 9% had abdominal pain. 4% of our patients had GI symptoms as the first presenting symptoms without other associated respiratory symptoms or fever. Therefore, it is important to consider COVID-19 infection in children presenting with GI symptoms with early testing, promoting patient and staff safety.

GI symptoms can be associated with severe COVID-19 in children. A multicenter study of 101 pediatric inpatients described patients presenting with GI symptoms had higher CRP, procalcitonin, ferritin values and admission to PICU (15). The levels of CRP had an expectedly high predictive value as they reflect the activity of an inflammatory process (18). In our cohort, children with GI symptoms were more likely to have fever (*P* < 0.001) and cough (*P* = 0.004). Abdominal pain was associated with elevated CRP (*P* = 0.037), diarrhea and vomiting were associated with elevated procalcitonin (*P* = 0.034 and 0.002 respectively). However, we did not observe increased PICU admissions in children with GI symptoms (*P* = 0.57). In addition, children with elevated liver enzymes were more likely to have fever (*P* = 0.021). Therefore, children with GI symptoms and liver injury in our study were more likely to have abnormal inflammatory markers and display a more severe COVID-19 infection.

Previous studies performed in the United Arab Emirates demonstrated an increased risk for men of all ages to require intensive care (*p <* 0.01). Males were more likely to have at least moderate disease severity (*p* = 0.0083) and the risk of the non-mild COVID-19 was significantly higher (*p <* 0.05) in midlife adults and older adults compared to young adults (19). In addition, Al Zahmi et al. demonstrated that Caucasian or East-Asian COVID-19 patients tended to have a more severe disease despite a lower risk profile. In contrast to this, Middle Eastern COVID-19 patients had a higher risk factor profile, but they did not differ markedly in disease severity from the other ethnic groups (20). In this review, sex, age, ethnicity, BMI, and patient medical background medical disease did not predispose to the development of GI symptoms.

Approximately 1% of children with COVID-19 develop severe disease requiring admission to intensive care unit (9). Six (3%) of our patients required admission to PICU; of those 6 patients, 5 had background complex medical disease and 1 was obese. A metanalysis on 285,004 children with confirmed SARS-CoV2 infection, 9,353 (3.3%) had at least one underlying comorbidity, of which 5.4% were obese. Among 507 obese children, 64 had severe COVID-19 or required ICU admission, with a calculated risk of severity of 2.87 (95% CI 1.16–7.07) (21). Among the180 patients included in this study, 52 (29%) had background medical disease, 12 (7%) were obese. Among the 12 obese children, 1 patient developed severe COVID infection and required PICU admission. Despite this the outcome was very good with no reported mortality and only one patient developed long term complication.

The authors recognize a few limitations to this study. Firstly, this is a retrospective study and as such may be limited by inaccurate documentation. Secondly, some children with COVID-19 may not have been captured in this study despite the extensive testing and contact tracing within the United Arab Emirates. Thirdly, BMI and blood testing was not performed in all patients. Fourthly, this is a relatively small study with small sample size which may limit the power of conclusions drawn from this study. Fifthly, despite the low accuracy of the multiple regression model, it helped us to analyze statistically significant predictors and draw important conclusions about how changes in the predictor values are associated with changes in the response value. The supposed reason for the low R-squared value is the limit sample size and the high number of regressors. With an increasing number of regressors, we need an increasing amount of data to obtain reliable estimates. Finally, we also acknowledge that the study was performed at the initial time of the pandemic and there are new strains that have evolved after the study period.

This preliminary examination of GI manifestation of COVID-19 disease in children suggest that children do not always present with fever or cough. Vomiting, diarrhea and abdominal pain need to be considered as part of the screening assessment in children. Children with GI symptoms are likely to have fever, cough and raised inflammatory markers and therefore display a more sever clinical course. Although COVID-19 has a favorable clinical outcome in children, the importance of identifying pediatric cases is to reduce the spread of infection and identify high risk patients.

## Data Availability

The original contributions presented in the study are included in the article/Supplementary Material, further inquiries can be directed to the corresponding author/s.
